# Report of Deaths in Buffalo for the Month of October, 1855

**Published:** 1855-12

**Authors:** J. Root

**Affiliations:** Health Physician


					﻿ART. VII. — Report of Deaths in Buffalo for the month
'________________________________________ AGE.
5 C9 aj
■a i- o 5
-	q
P
DISEASES.	___________
•	co	I
$	«	-•	A	-2
S	A	ol	5	®	g
A	o	o	—	-	—	a
S	tn	b	£	,®
__________________	A X A
Apoplexy,...........................................   2	....	2	........
Asthma,............................................... 1	1
Atrophia.............................................. 1	j 2 j ” j
Abscess Pleuritic, ................................... 1	|
Abdominal Tumor,..................................... 1
Consumption,..........................................16	'll’	27	’4'2	'1'1
Cholera,.............................................. 1	j
“ Infantum,....................................... 9	i	3	0	1
Croup .................................................3	3	6	2	'1 1
Convulsions,.......................................... 3	5	g	2	3	2
Congestion of Lungs,.................................. 1	.	j	'	'	]
Disease of Liver,.................................... 1
Diarrhoea,.............................................5	”5' 10 Wi
Dysentery,............................................ 3	14	1 ..
Delirium Tremens,..................................... 1	j
Debility,.............................."’’‘”'"'"""1'	1. ”3’	4
Drowned,.............................................. 4	4
Dentition,............................................ 2	"5"	7	114
Erysipelas.............................................1	j	....
Fever Scarlet,........................................ 3	”3'	g	"	1	3 1
-	“ Nervous............................................... j	1
“ Typhoid,.......................................... 1	1	2	/
“ Intermittent,......................................... 1	1
“ Brain,............................................ 1	1	"	j
“ Bilious,.......................................... 1	1
Hydrocephalus,........................................ 1	"”	j	--	--	j
Haemorrhage Internal,................................. 1	’’’’	1
Inflammation of Bowels,............................... 2	2
Inflammation of Brain,.................................... j	1	”	j
Intemperance,......................................... 1	j	2	'
Measles,.......................................... ;	1 j 2	1
Marasmus,............................................. 2	2	4	I	1 2
Old Age,.............................................. 3	2	5
Pulmonary Apoplexy,....................................... j	j	' ’
Paralysis,............................................ 2	2
Premature Birth,...................................... 1	_	1	j
Scrofula,............................................. 1	””	j	1
Still-born,........................................... 2	2	4
Typhoid Pneumonia,........................................ 1	1
Ulcer,................................................ 1	2
Unknown............................................... 8	"g	14	3 5	2--
Whooping Cough,....................................... 2	....	2	1
Totals,.............................~84~	~58~ HIT 20 22 16 15
Nativities.—American,84. German,27. English,4. Irish, 11. Scotch, 2.
of October, 1855. By J. Root, M. D., Health Physician.
AGE.
_	. s'
lOOiOOOOOOOOO'g’Dg
I'|llllllllll7*b“
p
--------------------------------K- -------------------
aj I I o ©	©	©	©	©	©	' ©	©	©	©’ aJ ©	| ©
©c_2P“^-2n<J>~4,'~rJS"r2gr^igp©.5iprJ2^®~®=rS|H
5;Ljz©”©Jx©rt©J:©rt©J:i'Jx©^i^©4?“!a©”^-S^
................... 1................... 1............
1	’’ ’’ ’’ ’’ ’’.. ’.'...'.'. " **.......’’ '.’ ’’ ’.’.’ " ..
......................... 1...........................
2	.. 1 1 1 .. .. 1 1 2 1 .. 1 2 1 3 1 .. 1..........
.. 1............ ........
.....
2 ”....................................
.h:
'i _i:::::::::::::::::::::: 'i:::: .k: ’i::::::::::::::::::::::
:::P::::::
’’	’’ .. ’’ 2 ’’i.. ’’ ’.'. i --'.’’.'.’.’ ” ’.’	’’ i
’........ i’................. .................
...... ]..............................................
......................... 1...................................
::::::..:: ":: :::: ”::..:::: i.. ::::....::::::::::....::::
«
’' i	'..’	’ * *.i..............\ *.. ’' ” ’" ’ *.. \..
.... i.......i.......................................
” " ’’	'.'. ." ’’ i ’’ ” ’’ 1.. ...’...’. .’ ’’ ..'.
1.......... ........................................
’’	'.. ’’ i i .. i i ’’i * ’’ ’’.'
.. i.............. ....................................... ....
.................. 1........ 1.......................
’. ..’.. ’. ’’ ’’ ’’ ’’’’	’. i .... .’ ’’ ” ’.....
..................... 1..............................
- 1 1 1.................................................... i..
1...................................................
10^!"4”2U ’OlTlll’O 3 ~4 ~6 " 4 ~6 ~T ~5 ~0 1 2 “lU) “0 ~1 ~0 “0 [ ~1
Hollander, 1. Canadian, 3. Swiss, 1. Col’d, 1. Country not given, 8.—Total, 142.
				

